# Discovery of Rare Variants via Sequencing: Implications for the Design of Complex Trait Association Studies

**DOI:** 10.1371/journal.pgen.1000481

**Published:** 2009-05-15

**Authors:** Bingshan Li, Suzanne M. Leal

**Affiliations:** Department of Molecular and Human Genetics, Baylor College of Medicine, Houston, Texas, United States of America; University of Alabama at Birmingham, United States of America

## Abstract

There is strong evidence that rare variants are involved in complex disease etiology. The first step in implicating rare variants in disease etiology is their identification through sequencing in both randomly ascertained samples (e.g., the 1,000 Genomes Project) and samples ascertained according to disease status. We investigated to what extent rare variants will be observed across the genome and in candidate genes in randomly ascertained samples, the magnitude of variant enrichment in diseased individuals, and biases that can occur due to how variants are discovered. Although sequencing cases can enrich for casual variants, when a gene or genes are not involved in disease etiology, limiting variant discovery to cases can lead to association studies with dramatically inflated false positive rates.

## Author Summary

One focus of human genetics is localizing genes that are involved in the etiology of complex diseases. Although emphasis has been placed on mapping common variants, recent studies have demonstrated that rare variants also play an important role in complex trait etiology and their identification should have a greater impact on risk assessment, disease prevention, and treatment due to their large genetic effects. Genome-wide association studies are used to identify common variants by genotyping tagSNPs that are proxies for common causal variants. This study design is not adequately powered for association studies of rare variants; instead, causal variants must be identified and then analyzed. With the development of sequencing technologies, it is feasible to sequence candidate genes and, soon, entire genomes to obtain data on rare variants for complex disease association studies. We investigated several questions that are germane to the discovery of rare variants within a sample; for example, proportion of variants discovered within a random sample and enrichment of causal variants within samples of cases compared to a random sample. We also demonstrate that when an excess of cases are sequenced to discover variants and the remaining samples are genotyped, this design strategy can lead to inflated false positive rates.

## Introduction

Genome wide association studies using indirect mapping have been successful in localizing genes which are associated with complex diseases when common variants are the underlying cause of disease etiology [1],[2]. Strong linkage disequilibrium (LD) between tagSNPs and underlying causal variants makes it feasible to use indirect mapping. To detect associations with rare variants the indirect LD mapping will be low-powered due to weak correlations between common tagSNPs and rare causal variants. In order for variants to be highly correlated they must have similar allele frequencies and the maximum *r^2^* value of 1 can be obtained only when the two loci have equal minor allele frequencies [3]. Therefore for associations with rare variants it is necessary to perform direct mapping and rare variants within a sample must first be identified. Sequencing of candidate genes or entire genomes is the optimal way to identify rare variants. A number of studies have successfully used the approach of sequencing candidate genes to carry out association studies for several complex traits [4]–[11]. There is emerging interest in association studies of rare variants and it is hypothesized that rare variants are more likely to be functional than common variants [12]. Although the genotypic RRs of rare causal variants are not elevated enough to produce familial aggregation, they are considerably higher than the genotypic RRs of common variants which are involved in complex disease etiology [13]. New sequencing technologies (e.g., Illumina Solexa, ABI SOLiD, and Roche 454) [14] have greatly reduced the cost of generating large amounts of sequencing data. This advancement has enabled the launch of the 1,000 Genomes Project, which will sequence at least 1,000 genomes from 10 different ethnic backgrounds. The project's goals include providing a detailed catalog of human variants to facilitate the identification of disease causing variants [15]. Even if the assumption holds that all variants present in a sample can be successfully identified through sequencing (i.e., no false negatives), variants may not be observed within a sample solely due to the randomness of sampling. Therefore we investigated three main questions for a randomly ascertained sample of 100–1,000 individuals for variants with frequencies between 0.01% and 1.0%: (1) What is the probability that a variant will be discovered at a specific site? (2) What proportion of variants will be uncovered across the entire genome? and (3) What is the probability that a certain proportion of variants will be discovered in a gene? It was also investigated to what extent carrying out variant discovery in cases increases the probability of uncovering causal variants compared to when variant discovery is performed in a randomly ascertained sample. Due to the high cost of sequencing, it may be attractive to sequence a subset of a sample of individuals for variant discovery and genotype the remaining samples, in particular to sequence an excess of cases due to their potential enrichment for causal variants. It was investigated how such strategies impact type I error.

## Results

### Discovering Rare Variants at a Specific Site and across the Genome

The probability of observing a variant at least once at a specific site was calculated for variants with a frequency of 1%, 0.5%, 0.2%, and 0.1% in sample sizes of 100–1,000 individuals (Table 1). The probability of observing a variant one or more times at a unique site in a sample is equivalent to the average proportion of variants discovered across genomes when linkage equilibrium is assumed among variants with equal frequency and discovery is carried out using the same number of individuals. It is observed that for variants with 1% frequency, even if the genomes of only 100 individuals are sequenced, 0.866 of all variants present in the population from which the sample was ascertained will be discovered. If the variant frequency is 0.1%, sequencing 100 genomes will only uncover 0.181 of all variants within the population; if the sample size is increased to 1,000 genomes, the proportion of variants discovered increases to 0.865 (Table 1).

**Table 1:** The proportion of variants identified in samples randomly ascertained from the population.

Frequency	*N* = 100	*N* = 200	*N* = 500	*N* = 1,000
0.001	0.181(0.012)	0.330(0.015)	0.632(0.015)	0.865(0.011)
0.002	0.330(0.015)	0.551(0.016)	0.865(0.011)	0.982(0.004)
0.005	0.633(0.015)	0.865(0.011)	0.993(0.003)	1.000(2.1E-4)
0.01	0.866(0.011)	0.982(0.004)	1.000(2.1E-4)	1.000(1.4E-6)

The proportion of variants discovered assuming linkage equilibrium and their standard deviations (shown in parentheses) in samples of *N* = 100, 200, 500, and 1,000 individuals for variants with equal population frequencies of 0.001, 0.002, 0.005, and 0.01. Although the mean proportions of variants discovered are not dependent on the number of variants in the genome, the standard deviations will vary depending on the number of variants. The standard deviations shown are for *M* = 1,000 variants. All proportions of variants discovered displayed as 1.0 were rounded up and their actual values are between >0.999 and <1.0.

### The Probability of Observing Rare Variants with Equal Frequencies within a Gene in a Randomly Ascertained Sample

**Figure 1:**
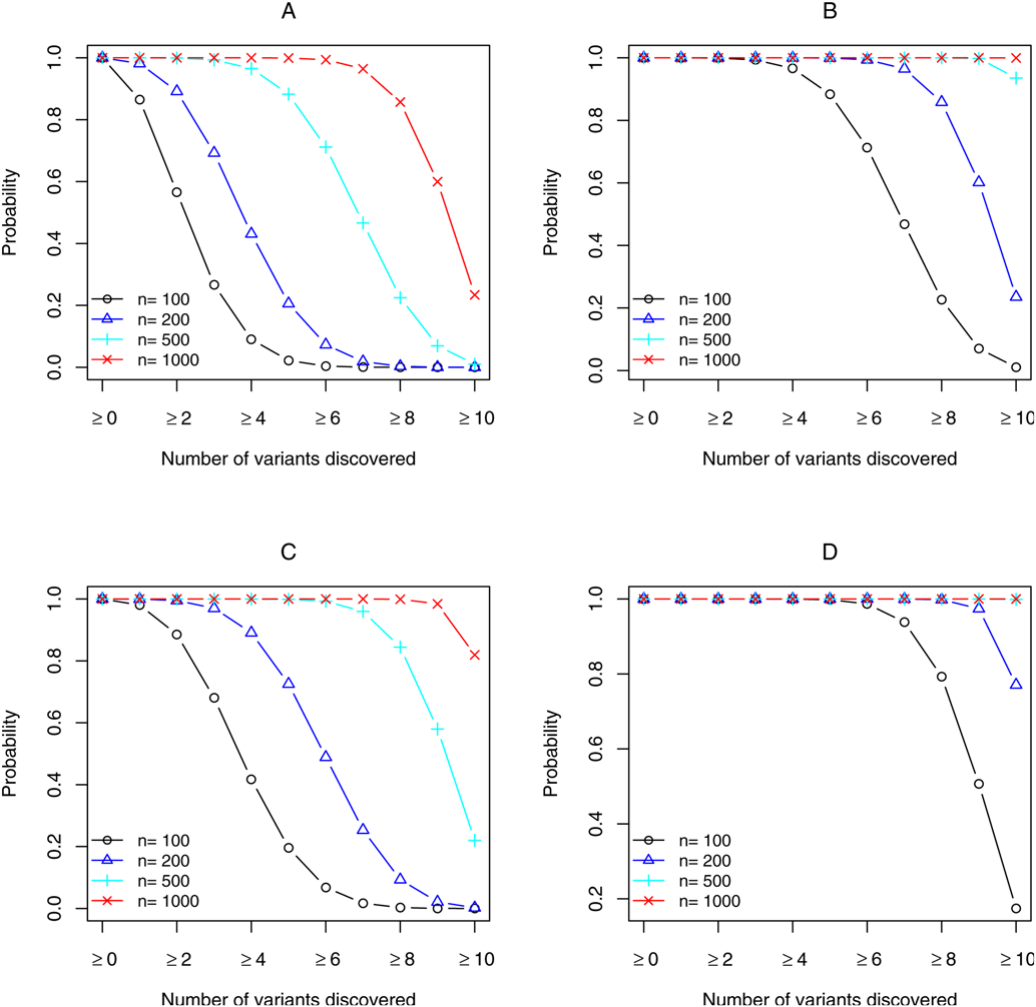
The probabilities of variant discovery for a gene with ten rare variants that have equal population frequency and reside on separate haplotypes. Panels (A and B) display the probability of variant discovery in randomly ascertained samples when each of the variants has a population frequency of 0.001 (Panel A) or 0.005 (Panel B). Panels (C and D) display the probability of discovering causal variants in samples of cases when each of the ten rare variants has a genotypic RR = 2.0 under a dominant model and population frequency of 0.001 (Panel C) or 0.005 (Panel D).

For a randomly ascertained sample, it was examined what proportion of variants within a gene will be discovered if they are assumed to be independent (i.e., in linkage equilibrium). For example if a gene with 10 variants each with 1.0% frequency is sequenced in a sample of 100 individuals, the probability is 0.860 that ≥8 variants and 0.237 that all 10 variants from the population will be identified. If the sample size is increased to 1,000 individuals, the probability that all variants will be discovered is close to 1.0. If the 10 variants within the gene have a frequency of 0.1%, then sequencing the gene in 100 individuals will uncover ≥8 variants with probability 3.7×10^−5^ and increasing the sample size to 1,000 individuals will identify ≥8 variants with probability 0.857 and all variants with probability 0.234 (Table 2 and Figure 1A).

**Table 2:** The probability of identifying rare variants with equal frequencies within a gene in samples of randomly ascertained individuals.

*M*	Frequency	*N* = 100			*N* = 200			*N* = 1,000		
		50%	80%	1.00%	50%	80%	100%	50%	80%	100%
10	0.001	0.0220	3.7E-5	3.8E-8	0.2060	0.0032	1.5E-5	0.9992	0.8571	0.2340
	0.005	0.8836	0.2265	0.0103	0.9992	0.8584	0.2354	1.0000	1.0000	0.9996
	0.01	0.9993	0.8600	0.2373	1.0000	0.9994	0.8343	1.0000	1.0000	1.0000
20	0.001	0.0012	3.1E-9	1.5E-16	0.0863	2.2E-5	2.3E-10	1.0000	0.8770	0.0547
	0.005	0.9266	0.0903	0.0001	1.0000	0.8786	0.0554	1.0000	1.0000	0.9991
	0.01	1.0000	0.8805	0.0563	1.0000	1.0000	0.6961	1.0000	1.0000	1.0000

The probability of discovering at least 50%, 80%, and 100% of variants within a gene with *M* = 10 and 20 variants in linkage equilibrium with population frequencies of 0.001, 0.005, and 0.01 are displayed for samples of *N* = 100, 200, and 1,000 individuals. All probabilities of identifying rare variants that are shown as 1.0 were rounded up and their actual values are between >0.9999 and <1.0.

For rare variants within genes a more realistic assumption is that they reside on separate haplotypes and are not in linkage equilibrium [16],[17]. If rare variants do not lie on the same haplotype with other rare variants, although the variants are in complete LD (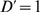), the correlation is extremely low (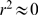). Therefore there is little difference in the results when it is assumed that the variants are on separate haplotypes (Table S1) or that they are in linkage equilibrium (Table 2). There is also a possibility that the rare variants are on the same haplotype and in this situation the correlation between the two variants is high and *r^2^* = 1 when the two rare variants have equal frequency. However, the probability that two rare variants within a gene occur on the same haplotype is extremely low and decreases with decreasing variant frequency.

### Enrichment of Causal Rare Variants with Equal Frequencies in Cases

If rare variants confer risk of being diseased, there is an enrichment of rare causal variants in cases. The magnitude of increase in frequency of causal variants in cases is dependent on variant frequency, genotypic RR and disease model (Figure 1 and Table 3). The relative increase is defined as the ratio of the frequency of causal variants in cases compared to their frequency in a randomly ascertained sample. If there is no difference between variant frequencies in cases and the reference group of randomly ascertained individuals, the relative increase is 1.0. If a gene has 10 causal variants each with a genotypic RR = 2.0 and a frequency of 0.1% in the general population, under a dominant model there is a 1.96 relative increase in frequency of variants in cases compared to a sample of randomly ascertained individuals; if the genotypic RR is increased to 5.0, the relative increase almost doubles to 4.63 (Table 3). The relative increase in frequency of causal rare variants with the same genotypic RR and population variant frequency is greatest under the multiplicative model, followed by the additive and dominant models with slightly smaller relative increases. For the recessive model, the relative increase for identifying causal variants in cases is very modest; for a gene with 10 casual variants each with a frequency of 0.1% and a genotypic RR of 5.0, the relative increase in frequency of causal variants is 1.04 (Table 3). Due to the enrichment of causal variants in cases when the underlying genetic model is multiplicative, additive or dominant, sequencing causal genes in cases compared to sequencing a randomly ascertained sample can substantially increase the probability of causal variant discovery (Figure 1).

**Table 3:** The relative increase in frequency of causal variants in samples of cases compared to samples of randomly ascertained individuals.

RR	Frequency^a^	Multiplicative			Dominant			Additive			Recessive		
		5	10	20	5	10	20	5	10	20	5	10	20
2	0.001	1.99	1.98	1.96	1.98	1.96	1.92	1.99	1.97	1.94	1.00	1.01	1.02
	0.002	1.98	1.96	1.92	1.96	1.92	1.85	1.97	1.94	1.89	1.01	1.02	1.04
	0.005	1.95	1.90	1.82	1.91	1.82	1.68	1.93	1.86	1.75	1.02	1.05	1.09
5	0.001	4.90	4.81	4.63	4.81	4.63	4.32	4.83	4.67	4.38	1.02	1.04	1.08
	0.002	4.81	4.63	4.31	4.63	4.32	3.81	4.67	4.38	3.91	1.04	1.08	1.15
	0.005	4.55	4.17	3.57	4.18	3.60	2.84	4.25	3.71	3.00	1.10	1.19	1.35

a Frequency of variants within the population.

The relative increase of causal variant frequency is shown for 5, 10, and 20 causal variants, each with equal population frequencies of 0.001, 0.002, and 0.005 and genotypic RR of either 2 or 5 under a multiplicative, dominant, additive, and recessive model. The calculations were carried out under the assumption that the causal variants reside on separate haplotypes.

### Rare Variants Discovery in Randomly Ascertained Samples and Cases for Variants with a Mixture of Frequencies

**Figure 2:**
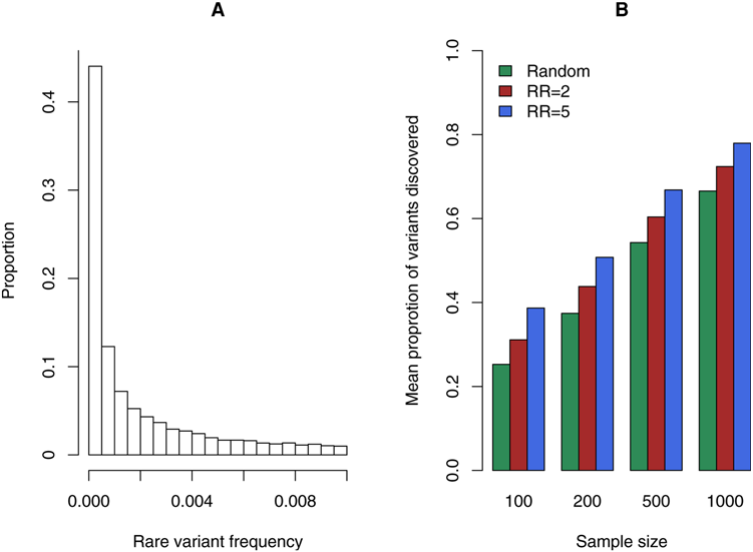
Distribution of the frequencies of rare variants and the average proportion of rare variants discovered in randomly ascertained and case samples. Data were generated using coalescent simulation under the neutral Wright–Fisher model with a scaled mutation rate θ = 4. Panel (A) displays the distribution of rare variants with frequency ≤0.01 for 100 haplotype pools each with 10,000 haplotypes. Panel (B) displays the mean proportion of variants discovered for randomly ascertained samples and for case samples of *N* = 100, 200, 500, and 1,000 individuals. Results are based upon 10,000 replicates. Case samples were generated with 50% of rare variants randomly chosen to be causal each with a genotypic RR of 2 or 5 under the additive model.

Although results assuming equal variant frequencies are easy to interpret, they are not realistic. Therefore variant discovery was also investigated using a more realistic distribution of rare variant frequencies by generating the data using coalescent simulation. Haplotype pools were generated under a neutral Wright–Fisher model where the scaled mutation rate 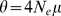 was set to 4. To reduce the impact of randomness 100 haplotype pools were generated with each pool containing 10,000 haplotypes. Since the interest in this study is on rare variants, only those variants with frequency ≥1% were investigated. The density of rare variants which were generated using coalescent simulations is shown in (Figure 2A). The distribution of rare variants is dominated by very low frequencies; for example, 86% of rare variants have frequencies <0.5%. When sample size is small, the majority of the rare variants are not observed in samples which are randomly ascertained from the population (Figure 2B). For example, only 25% of the rare variants are observed in a sample of 100 randomly ascertained individuals. When the sample size increases to 1,000 individuals, the proportion of discovered variants increases to 67%; even with this large sample size 33% of the variants are not observed due to the large proportion of very rare variants (Figure 2). Since the frequency distribution of variants is independent of the scaled mutation rate θ in coalescent simulations, the proportion of variants discovered is approximately the same for different θ values (data not shown). To investigate variants discovery in cases, half of the rare variants are randomly chosen to be causal and it is assumed that all causal variants have the same genotypic RR and the genetic model is additive (see Methods). When a sample of cases is sequenced to discover rare variants, the proportions of variants observed increase compared to when a randomly ascertained sample is sequenced (Figure 2B). For example, when the causal variants have a genotypic RR of 2.0, 31.1% of rare variants were observed in 100 cases and when the genotypic RR is increased to 5, this proportion increases to 38.7%, while for a randomly ascertained sample only 25.3% of the variants were observed. Even for a large genotypic RR of 5 the increase in the proportion of rare variant discovery is not dramatic compared to when variant discovery is carried out in a randomly ascertained sample (Figure 2). This is due to the fact that for coalescent simulations very rare variants dominate and the frequency of each rare variant in cases is still very low since this frequency is roughly the population frequency of the rare variant times its genotypic RR (see Table 3).

### Type I Error for Rare Variants with Equal Frequencies

Due to the increased probability of detecting rare causal variants in cases, it may be tempting to carry out discovery of variants in cases and then genotype these variants in controls. We considered the situation where variant identification is carried out in cases via sequencing in a gene that is not involved in disease etiology and these variants are genotyped in controls. The first scenario considered is for a gene that has a fixed number of variants with equal frequencies in the population. When the test of association is carried out on the sample of cases and controls, type I error rates are inflated unless the case sample size is sufficiently large (Table 4). For example, for a sample of 100 cases and 100 controls, the false positive rate is 0.067 at α level of 0.05 for a gene with 10 variants each with a frequency of 0.001; the false positive rate increases to 0.257 for a gene with 20 variants. We also examined the type I error rate when a definite number of variants are observed in cases, since in reality the true number of variants within a gene is unknown. If variant discovery is carried out in 100 cases and 5 variants are discovered each with a population frequency of 0.1% and these 5 variants are genotyped in 100 controls, the probability of rejecting the null hypothesis of no association is 0.38 (Table 5). When variants were identified in both cases and controls, the type I error rate is well controlled for varying sample sizes, number of variants and frequencies (Tables 4 and 5). For the two situations described where variant discovery is carried out in cases, type I error can be even more inflated if two or three times the number of controls to cases are genotyped (data not shown). Type I error inflation also occurs when individuals with either high or low quantitative trait values are sequenced to identify rare variants and individuals with quantitative trait values from the other end of the spectrum are genotyped. Type I error remains inflated, if variant discovery is carried out in a subset which contains a greater proportion of cases than controls or disproportionate numbers of individuals from one end of the quantitative trait spectrum (data not shown).

**Table 4:** False positive rates for association studies when variant identification is carried out in only cases or in both cases and controls for gene(s) with a fixed number of neutral variants.

		*M* = 10			*M* = 20			*M* = 30		
Discovery Sample	*N*	0.001	0.002	0.005	0.001	0.002	0.005	0.001	0.002	0.005
Cases Only	100	0.067	0.140	0.115	0.260	0.346	0.235	0.447	0.562	0.374
	200	0.144	0.132	0.055	0.350	0.273	0.083	0.553	0.430	0.113
	500	0.107	0.055	0.048	0.219	0.079	0.049	0.334	0.104	0.049
	1,000	0.057	0.047	0.050	0.078	0.048	0.050	0.102	0.048	0.050
Cases and Controls	100	0.039	0.043	0.051	0.042	0.050	0.050	0.046	0.050	0.050
	200	0.045	0.049	0.049	0.049	0.048	0.050	0.048	0.050	0.050
	500	0.050	0.049	0.050	0.049	0.050	0.050	0.049	0.050	0.050
	1,000	0.051	0.050	0.050	0.050	0.050	0.050	0.050	0.050	0.050

Results are shown for gene(s) with *M* = 10, 20, and 30 neutral variants with equal population frequencies of 0.001, 0.002, and 0.005, for *N* = 100, 200, 500, and 1,000 cases, and an equal number of controls. The assumption is made that the variants reside on separate haplotypes. The upper panel shows the false positive rates when only cases are used for variant discovery and the discovered variants are genotyped in controls. The lower panel shows the false positive rates when both case and controls are sequenced to discover rare variants. Analyses were carried out using the Cochran–Armitage test for trend (see Methods). The false positive rates were evaluated for an α = 0.05 and based upon 100,000 replicates.

**Table 5:** False positive rates for association studies when a definite number of neutral variants are identified only in cases or when variant discovery is carried out in cases and controls.

		*M* = 5			*M* = 10		
Discovery Sample	*N*	0.001	0.002	0.005	0.001	0.002	0.005
Cases Only	100	0.389	0.211	0.087	0.864	0.584	0.186
	200	0.217	0.111	0.045	0.579	0.245	0.060
	500	0.080	0.046	0.047	0.175	0.058	0.048
	1,000	0.047	0.047	0.051	0.060	0.047	0.050
Cases and Controls	100	0.040	0.038	0.047	0.050	0.052	0.051
	200	0.040	0.045	0.047	0.053	0.049	0.050
	500	0.042	0.051	0.048	0.05	0.048	0.049
	1,000	0.051	0.052	0.050	0.052	0.050	0.050

The false positive rates are displayed for when *M* = 5 or 10 neutral variants with equal population frequencies of 0.001, 0.002, and 0.005 were discovered in cases (upper panel) or in both cases and controls (lower panel) for *N* = 100, 200, 500, and 1,000 cases with equal number of controls. It is assumed that each rare variant resides on a separate haplotype. Analyses were carried out using the Cochran–Armitage test for trend (see Methods). The false positive rates were evaluated for an α = 0.05 and based upon 100,000 replicates.

### Type I Error for Rare Variants with a Mixture of Frequencies

Coalescent simulation was used to generate rare variant data as described in the Methods section. When simulation was used to mimic the situation where variants discovery is performed in cases and the controls are genotyped for those discovered variants, inflated type I error at α level of 0.05 was observed for different θ values when the locus analyzed is neutral (Table 6). Since the length of a simulated locus is proportional to the θ value when the size of a haplotype pool is fixed, the number of rare variants and their total variant frequency increase with larger *θ* values and represent a longer locus or multiple loci (Table 6). Therefore the type I error rate increases with increasing *θ* values. For example, the type I error rate is 0.124 for *θ* = 4 and a sample of 100 cases and 100 controls; it increases to 0.325 when *θ* = 12 for the same sample size of cases and controls (Table 6). For all *θ* values, the type I error rate decreases with increasing sample size and when the sample size is 1,000 cases and 1,000 controls, the type I error rate is very close to the α value of 0.05. For all sample sizes and *θ* values, when both cases and controls were used to discover rare variants, no inflated type I error rates were observed (Table 6).

**Table 6:** False positive rates for rare variant association studies when variants are identified only in cases or in both cases and controls for variants with a mixture of frequencies.

				Number of Cases^a^			
Discovery Sample	*θ*	Number of variants^b^	Frequency^c^	100	200	500	1,000
Cases Only	4	21.0	0.0403	0.124	0.094	0.066	0.056
	6	31.5	0.0628	0.178	0.122	0.076	0.062
	8	41.9	0.0835	0.228	0.146	0.085	0.066
	12	61.7	0.1160	0.325	0.205	0.106	0.075
Cases and Controls	4	21.0	0.0403	0.048	0.050	0.050	0.050
	6	31.5	0.0628	0.050	0.047	0.049	0.049
	8	41.9	0.0835	0.048	0.048	0.051	0.051
	12	61.7	0.1160	0.049	0.049	0.049	0.051

a The number of controls is equal to the number of cases.

b Number of rare variants with frequency ≤1% observed per haplotype pool averaged over 100 haplotype pools.

c Total frequency of rare variants with frequency ≤1% observed per haplotype pool averaged over 100 haplotype pools.

Coalescent simulations with scaled mutation rates *θ* ranging between 4 and 12 were used to generate genotype data for rare variants with frequencies between 0.0001 and 0.01 for samples of *N* = 100, 200, 500, and 1,000 cases. The false positive rates are displayed when variant discovery is carried out in only cases via sequencing and the discovered variants are genotyped in controls (upper panel) and when both cases and controls are sequenced to discover rare variants (lower panel). Analyses were carried out using the Cochran–Armitage test for trend (see Methods). The false positive rates were evaluated for an α = 0.05 and based upon 100,000 replicates.

Since the data were generated using the neutral Wright–Fisher model without recombination, it was investigated whether it is valid to use larger *θ* values for the situation where multiple loci are combined to be analyzed. Two loci for each individual were obtained from two independent haplotype pools each generated using a scaled mutation rate *θ* = 6. These results are very similar to those obtained when individual genotypes were sampled from a single haplotype pool generated using *θ* = 12 (data not shown). This is an indication that recombination between loci should have little impact on the results.

## Discussion

The 1,000 Genomes Project will bring to light a wealth of information on human variation and should be able to capture a vast majority of variants with a frequency of >1%. A detailed catalog of variants should aid association studies of complex traits to study variants which range from common to rare. It is hypothesized that rare causal variants for complex diseases are usually found in the frequency range between 0.1% and 1%, although the boundaries are not absolutely defined [13]. The 1,000 Genomes Project will also identify very rare variants (e.g., frequency <0.5%), however, the study's ability to discover a substantial proportion of very rare variants will be dependent on whether or not very rare variants are shared across multiple populations, because individual ethnic groups which are included in the project will have a limited sample size, ∼100 individuals. Many rare variants have occurred in recent human history and therefore they may not be shared among different populations. Thus the 1,000 Genomes Project currently does not have an adequate sample size to provide a comprehensive catalog of very rare variants which could be selected for genotyping in association studies of complex traits.

Although assuming equal variant frequencies is not realistic, it is easier to interpret these results than when a mixture of variant frequencies is used. To also investigate a more realistic situation where variants have a mixture of frequencies, coalescent simulation was used by generating haplotype pools under a neutral Wright–Fisher model with the assumption of no recombination. The simulation of haplotypes which reflect evolutionary history of human populations has been well researched and a neutral Wright–Fisher model is commonly used. For genes the impact of recombination is negligible due to gene length and genome-wide surveys [18] have shown that recombination events occur unevenly across the human genome, and preferentially transpire outside gene boundaries. However, in reality genetic regions may display different distributions of variant frequencies than those obtained using coalescent simulation and therefore rare variant discovery may exhibit different results.

If it is believed that very rare variants contribute to disease etiology, sequencing of the study sample will be necessary to identify them. Although causal variants will be enriched in case samples, most genomic regions which are sequenced will not be involved in disease etiology. If cases are sequenced and the identified rare variants are genotyped in controls, this can lead to an increase in type I error, with the estimate of the OR being >1.0. The increase in type I error can also occur if the controls are sequenced and the cases are genotyped since the genes are not causative; in this situation the estimate for the OR will be <1.0. For situations where different proportions of cases and controls are sequenced and the remaining samples are genotyped, type I error may also be inflated. In a similar fashion, if to identify rare variants the exons of a gene are sequenced in cases and only those exons where rare variants were detected are sequenced in the controls, type I error can also be inflated. The differences in the variant frequencies between cases and controls are intrinsic to this study approach and cannot be controlled for by permutation. This inflation of type I error will not occur if the subjects that are used for variant discovery are not included in the association study. Whether or not an inflation of type I error occurs is dependent on the size of the initial sample which is sequenced, variant frequencies and the number of variants within the gene/genomic region. If the analysis is done on a specific gene/region, the level of type I error inflation is not monotonic with sample size or variant frequency as shown in Table 4. The type I error is a function of both the sample size and the difference in variant frequency between cases and controls. For small sample sizes, although the frequency difference between cases and controls is great the power to detect the difference is low due to sample size. On the other hand, for large sample sizes the variant frequency difference between cases and controls is small and the power to detect these small differences is also low, even though the sample size is large. Therefore the greatest inflation of type I error occurs for a moderate sample size and the exact sample size depends on the population frequency of rare variants. Although a monotone decrease in type I error was observed with increasing sample sizes for the examples displayed in Table 5 and Table 6, monotonicity was violated when smaller sample sizes were analyzed (data not shown) demonstrating that monotonicity is not always the rule. Since neither the frequencies of variants in a population nor the number of variants within the gene/genomic region are known a priori, it is not possible to elucidate whether or not type I error has been inflated if variant discovery is carried out in a preponderance of cases.

Collapsing of genotypes was used for the association tests. It is also possible to analyze each variant separately, however for this approach to have sufficient power extremely large sample sizes will be necessary [12], with sample sizes increasing with decreasing variant frequencies and genotypic RRs. Power is particularly low when variants are either recent or de novo. Collapsing has been shown to be a powerful approach to analyze rare and very-rare variants [19] and therefore we used it in our analyses.

Since mutation rates are unlikely to vary in different populations, it might be tempting to use the data from the 1,000 Genomes Project as a reference control population for various studies of complex traits. However the aggregate frequencies of rare variants in a genomic region may vary greatly from one ethnic group to another [13],[20] due to different evolutionary histories including genetic drift and bottlenecks. There are a number of examples where rare causal variants (e.g., variants in the *CFTR*, *BRCA1*, and *BRCA2* genes) have higher frequencies within the Ashkenazi Jewish population compared to other European Jewish and non-Jewish populations [21],[22]. In addition to rare causal variants having varying frequencies within ethnic groups, rare neutral variants may also have diverse frequencies which can lead to an increase of type I error if population substructure is not adequately controlled [23]. In the study of rare variants, it is currently unknown if a consensus panel of controls can be used; for example, a European panel for complex trait association studies of Europeans and individuals of European descent, or if more stringent matching criteria are necessary. Additionally it has not been investigated if implementing current statistical methods; for example, principal components analysis [24] using common variants will adequately control population substructure when analyzing rare variant data.

Studies of rare variants for complex traits are beginning to emerge and in the near future a large number of studies will be carried out for a variety of common diseases. Although there are many challenges in understanding the involvement of rare variants in complex disease etiology, one benefit from the study of rare variants compared to common variants is that rare variants have higher genotypic RRs, not only making it easier to implicate them in complex disease etiology but also the identification of rare variants should have a greater impact on risk assessment, disease prevention and treatment [13].

## Methods

### Probability of Observing Rare Variants That Are in Linkage Equilibrium in a Randomly Ascertained Sample

Let the number of variants equal 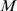 and each variant site has two alleles 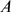 and 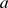 with a minor allele frequency of 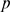. It is assumed that 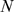 individuals are sampled from a population where Hardy-Weinberg Equilibrium (HWE) holds. If all variants in a sample are successfully identified via sequencing (i.e., no false negatives), the probability of observing a specific variant one or more times within a randomly ascertained sample is 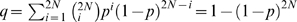. Under the assumption of linkage equilibrium (LE) between variants, the probability of discovering 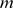 or more different variants in the randomly ascertained sample is 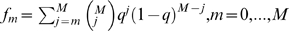.

### Probability of Observing Rare Variants That Are on Separate Haplotypes in a Randomly Ascertained Sample

Usually rare variants are relatively young and reside on separate haplotypes within a candidate gene or small genomic region and additionally within small genomic regions there is little or no recombination [16],[17]. We assumed that within a gene rare variants are on different haplotypes and there is no recombination. In this situation, rare variants are not independent. Although they are in complete LD (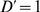), the correlation between the variants is extremely low; that is, 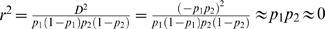 where 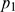, 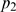 are the frequencies of two rare variants. In this situation, under the assumption of HWE, the number of variants with equal frequency observed in a sample follows a multinomial distribution with parameter 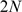 and 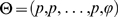 where 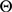 is a vector of size 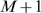 and 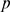 and 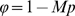 are the frequencies of each rare variant bearing haplotype and non-rare variants bearing haplotype, respectively. A discrete Markov Chain (MC) was constructed to facilitate the calculation of the probability of observing 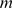 or more variants in a sample of 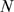 individuals or 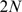 chromosomes. A sample of 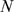 individuals can be treated as a realization of MC where the process is that 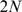 chromosomes are sampled one at a time assuming HWE. Let 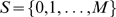 denote the state space and let 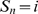 represent the event that 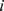 variants are observed at time *n*; that is, when *n* chromosomes are sampled. When sampling the *(n+1)^th^* chromosome, the transition from state 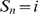 to 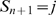 occurs when 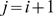 or 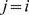, corresponding to when one or zero additional variants are observed. The transition matrix is
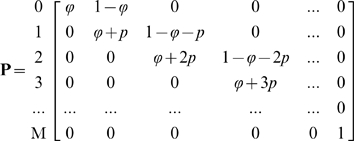
where the column outside of the matrix are the states. Let the row vector 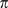 of length *M+1* denote the probability distribution of observing *k* of *M* variants where *k* = *0,1,…,M*. Then the initial probability vector 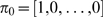, denoting that no variants are observed when no chromosomes are sampled. The probability distribution of the number of variants observed in a sample of 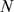 individuals is 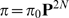 and the probability of observing 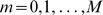 or more variants is 
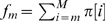
, 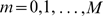.

### Probability of Observing Variants That Are on Separate Haplotypes in a Sample of Cases

Suppose each of 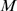 causal variants resides on a separate haplotype and independently increases disease susceptibility (i.e., allelic heterogeneity model). Denote the haplotypes as 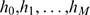 with frequency 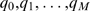 where 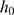 represents the non-rare variants bearing haplotype and 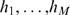 are high risk haplotypes carrying rare variants. Let the penetrance of genotype 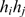 be represented by 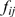; that is, 
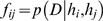
. Define the marginal haplotype penetrance of 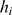, denoted by 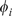, as the probability of being diseased if an individual carries the haplotype 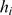; that is, 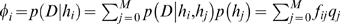 under the assumption of HWE. The frequency of haplotype 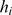 in cases is 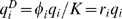 where 
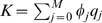
 is the disease prevalence and 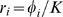 is the ratio of relative increase of variant frequency in cases compared to a randomly ascertained sample. When it was assumed that all variants have the same genetic effect, denote the penetrances of genotype 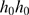, 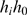 and 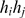 as 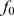, 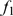 and 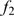 respectively and define the genotypic RR 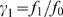 and 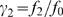. For multiplicative, additive, dominant, and recessive models, the RRs satisfy 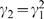, 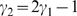, 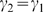 and 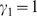, respectively.

### Effect on Type I Error of Association Studies When Variant Discovery Is Only Performed in Cases

Suppose rare variants are discovered in cases via sequencing and then those variants are genotyped in controls. When the sequenced gene is not involved in the etiology of the disease under study, the variants observed in cases are independent of the cases status. Conditional on the fact that a variant with frequency 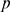 has been observed at least once in cases, the mean number of times the variant is observed in cases at the marker is 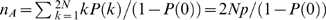 where 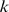 follows binomial (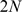, 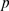) distribution for a sample of 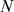 cases and the mean number of times that this variant is observed in the same number of controls is 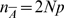. Since 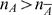, this may create false positive associations. Since analyzing variants individually is underpowered [12], it is advisable to collapse multiple variants across the 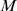 markers at the locus to increase power to detect associations [19]. In this study the locus with multiple rare variants is collapsed in such a way that the locus of one individual is coded using the number of rare variants this individual carries; in this way multiple variants are collapsed into a single number. The association test is used to test whether one or more rare variants are associated with the disease. Under the allelic heterogeneity model that multiple rare variants cause the disease independently, it is expected that a locus with more variants has a higher probability of being diseased and the Cochran–Armitage test for trend is natural to use where the ordered categories are the number of variants present at a locus. The false positive rates were evaluated using simulated data assuming rare variants reside on separate haplotypes. For simplicity it was assumed all haplotypes have the same frequency 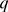 and 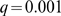, 0.002 and 0.005 were used as examples. For the scenario where the number of variants at a locus within the population is fixed, the genotype for each individual was constructed by randomly sampling two haplotypes and then was randomly assigned to either the case or control sample. This process was repeated until the desired sample size was obtained for both cases and controls. Only those variants which are observed in cases are analyzed in the case-control dataset. For the situation where both cases and controls are sequenced, the same procedure was performed except that variants observed in either cases or controls were included in the analysis. For the scenario where a definite number of variants are observed in cases, the genotype for each individual was constructed by randomly sampling two haplotypes of length 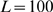 and then randomly assigned to either the case or control sample. This process was repeated until the desired sample size was obtained. Among the variants which were observed in cases, a total of either 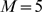 or 10 variants were randomly selected and then the 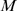 variants were analyzed in the case-control dataset. In order to perform the simulation for the situation where variant discovery is performed in both cases and controls, 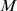 variants were randomly selected in the sample (cases and controls) and then the 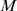 variants were analyzed in the case-control dataset. For each study the analyses were repeated 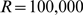 times and the type I error rate was estimated for the 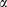 level of 0.05 by calculating the proportion of replicates with 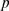 values 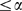.

### Rare Variants Discovery and Evaluation of Type I Error Rates for Variants with a Mixture of Frequencies Using Coalescent Simulation

Although the results are easier to interpret when all variants have equal frequency, rare variants usually have a mixture of frequencies at a locus within a population. To investigate the variant discovery and type I error rates in a more realistic scenario, coalescent simulator *ms* [25] was used to generate haplotype pools to represent a population. The scaled mutation rate of a locus, 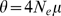, was set to values between 4 and 12 to represent different locus lengths, and 10,000 haplotypes were simulated for each haplotype pool assuming no recombination within the locus. To reduce randomness of coalescent simulation, 100 replicates were generated for each θ value. Since the interest is on rare variants, only variants with frequencies between 10×10^−4^ and 10×10^−2^ were analyzed. To generate a sample, a haplotype pool was randomly selected and all individuals' genotypes are formed by randomly pairing two haplotypes from this pool. For randomly ascertained samples, *N* individuals were selected from the haplotype pool. For case samples, an individual was assigned case or control status according to the penetrance of the genotype. It is assumed that not all variants are causal and 50% of variants are assigned to be causal with the same genotypic relative RR. The penetrace of the genotype follows an additive model and is calculated as m*γ_1_*ƒ_0_, where *m* is the number of rare causal variants the individual carries at the locus, *γ_1_* is the RR of the genotype carrying one causal variant versus the genotype carrying no causal variants and *γ_1_* = 2 and 5 were used. This process is repeated until the desired sample size was obtained. For either the randomly ascertained sample or the case sample, variant discovery is performed by examining each position of the locus and counting the number of variants observed at the locus in the sample.

For evaluation of type I error, an individual was generated by pairing two random haplotypes from a haplotype pool and was randomly assigned to case or control status. The process was repeated until the desired sample size was obtained. The type I error rates were estimated based on 100,000 replicates for an α level of 0.05 in the same manner as described in the previous Methods section for the scenario where the number of variants at a locus within the population is fixed.

## Supporting Information

Table S1 The probability of identifying rare variants with equal frequencies in samples of randomly ascertained individuals when the rare variants residue on separate haplotypes.
(0.05 MB DOC)
