# Supplementary material for: Discovery of Rare Variants via Sequencing: Implications for the Design of Complex Trait Association Studies
Source: PLoS Genet. 2009 May 15;5(5):e1000481. doi: 10.1371/journal.pgen.1000481 (PMC2674213; doi:10.1371/journal.pgen.1000481)
Supplement: Table S1 — The probability of identifying rare variants with equal frequencies in samples of randomly ascertained individuals when the rare variants residue on separate haplotypes. (0.05 MB DOC) [file pgen.1000481.s001.doc]

Table S1 The probability of identifying rare variants with equal frequencies in samples of randomly ascertained individuals when the rare variants residue on separate haplotypes

|  |  |  |  | N=100 | | |  | N=200 | | |  | N=1,000 | | |
| --- | --- | --- | --- | --- | --- | --- | --- | --- | --- | --- | --- | --- | --- | --- |
| M |  | Freq |  | 50% | 80% | 100% |  | 50% | 80% | 100% |  | 50% | 80% | 100% |
| 10 |  | 0.001 |  | 0.0215 | 3.3E-5 | 1.5E-8 |  | 0.2051 | 0.0030 | 1.4E-5 |  | 0.9992 | 0.8574 | 0.2335 |
|  |  | 0.005 |  | 0.8870 | 0.2227 | 0.0096 |  | 0.9993 | 0.8598 | 0.2328 |  | 1.0000 | 1.0000 | 0.9996 |
|  |  | 0.01 |  | 0.9994 | 0.8630 | 0.2321 |  | 1.0000 | 0.9994 | 0.8338 |  | 1.0000 | 1.0000 | 1.0000 |
|  |  |  |  |  |  |  |  |  |  |  |  |  |  |  |
| 20 |  | 0.001 |  | 0.0011 | 2.0E-9 | 1.1E-16 |  | 0.0845 | 1.8E-5 | 1.7E-10 |  | 1.0000 | 0.8777 | 0.0542 |
|  |  | 0.005 |  | 0.9326 | 0.0838 | 0.0001 |  | 1.0000 | 0.8819 | 0.0529 |  | 1.0000 | 1.0000 | 0.9991 |
|  |  | 0.01 |  | 1.0000 | 0.8874 | 0.0511 |  | 1.0000 | 1.0000 | 0.6943 |  | 1.0000 | 1.0000 | 1.0000 |

For a randomly ascertained sample of N=100, 200, and 1,000 individuals, the probability of discovering at least 50%, 80% and 100% of the variants within a gene with M=10 and 20 variants with population frequencies of 0.001, 0.005 and 0.01. All probabilities of identifying rare variants which are shown as 1.0 in the table were rounded up and their actual values are between >0.9999 and <1.0.
